# The tumor microenvironment as a key regulator of radiotherapy response

**DOI:** 10.3389/fimmu.2026.1776636

**Published:** 2026-03-11

**Authors:** Xinchi Ma, Na Zhang

**Affiliations:** Department of Radiation Oncology, Liaoning Cancer Hospital & Institute, Shenyang, China

**Keywords:** cancer, cancer-associated fibroblasts, combination therapy, hypoxia, immune suppression, radiotherapy resistance, VEGF

## Abstract

Radiotherapy (RT) remains a cornerstone of cancer treatment, yet its efficacy is often limited by tumor recurrence and resistance. Emerging evidence underscores the pivotal role of the tumor microenvironment (TME) in this process. RT-induced vascular damage exacerbates hypoxia, a key driver of resistance, while activation of cancer-associated fibroblasts promotes fibrosis and extracellular matrix remodeling that shield tumor cells. Furthermore, RT elicits a complex immune response, capable of both immunogenic cell death and fostering an immunosuppressive milieu enriched with regulatory T cells and myeloid-derived suppressor cells. We discuss the mechanisms through which these TME alterations, hypoxia, fibrotic signaling, and immune evasion, collectively contribute to RT resistance and recurrence. In this review, we summarize current knowledge on how RT remodels the TME, focusing on its dualistic impact on vascular integrity, stromal activation, and immune regulation. Finally, we outline the promising therapeutic strategies in overcoming TME-mediated resistance, including vascular normalization, targeting hypoxia-inducible factors, and combining RT with immunotherapies such as immune checkpoint blockade. Overall, a deeper understanding of TME dynamics post-RT is crucial for developing novel combination therapies to improve clinical outcomes.

## 1 Introduction

Radiotherapy (RT) remains a mainstay in the treatment of solid tumors, contributing to curative intent in over 50% of cancer patients (1). Historically, research in radiation oncology has centered on tumor-intrinsic responses to DNA damage, including cell cycle arrest, apoptosis, and mitotic catastrophe (2). However, accumulating evidence over the past two decades has revealed that the tumor microenvironment (TME)—comprising stromal cells, vasculature, immune infiltrates, and extracellular matrix (ECM)—plays an equally critical role in shaping therapeutic efficacy (3, 4). Ionizing radiation not only damages cancer cells but also profoundly reshapes the TME, triggering complex and often paradoxical responses that can either support tumor regression or promote therapeutic resistance (5, 6).

TME components such as cancer-associated fibroblasts (CAFs), tumor vasculature, and infiltrating immune cells dynamically interact with irradiated tumor cells through signaling networks involving hypoxia-inducible factors (HIF-1α), angiogenic mediators (VEGF), and immunoregulatory cytokines (TGF-β, IL-6) (7–9). These interactions modulate inflammation, fibrosis, immune suppression, and abnormal vascular remodeling—all of which may attenuate the long-term benefits of RT (10). Furthermore, clinical and preclinical studies have demonstrated that TME-driven feedback loops can foster tumor relapse, metastasis, and resistance to immune checkpoint blockade, underscoring the need to reframe RT not as an isolated modality but as a TME-modifying intervention (11). This review systematically examines how RT remodels the tumor microenvironment to promote or hinder therapeutic outcomes. This review also highlights combinatorial strategies targeting hypoxia, CAFs, and immune suppression to overcome radioresistance and improve clinical response.

## 2 Effects of radiotherapy on the tumor microenvironment

### 2.1 Effects of radiotherapy on the vasculature system

The tumor microenvironment encompasses the complex niche in which tumor cells coexist with a variety of stromal and immune components (12–14). Beyond malignant cells, the TME includes the surrounding vasculature, infiltrating immune cells, cancer-associated fibroblasts, a repertoire of cytokines and chemokines, and the ECM, all of which dynamically interact to influence tumor behavior and therapeutic response (15). The vascular structure within the TME is typically abnormal and disorganized. This abnormal vasculature contributes to tumor metabolic dysregulation, primarily characterized by hypoxia and acidosis, which in turn leads to resistance of tumor cells to radiotherapy (16). Tumor blood vessels often lack an intact basement membrane and proper pericyte coverage, making them significantly more permeable, leaky, and less responsive to radiation than normal tissue vasculature (17). Irradiation with high-dose irradiation (15–20 Gy) has been shown to induce irreversible vascular damage within 6 hours, subsequently promoting tumor cell apoptosis and necrosis (18). Importantly, the extent and nature of radiation-induced vascular remodeling are contingent upon multiple factors, including the total radiation dose administered, tumor size, anatomical location, and pathological stag (19, 20).

During radiotherapy, radiation induces endothelial dysfunction, which manifests as increased vascular permeability, basement membrane detachment, and endothelial apoptosis (21–23). These changes further promote vascular inflammation and fibrosis. Radiation-induced vascular injury is dose-dependent and results in decreased microvessel density and ineffective perfusion of tumor parenchyma, ultimately causing tumor necrosis and exacerbated hypoxia (24, 25). The subsequent hypoxic environment induces the expression of hypoxia-inducible factor-1α (HIF-1α), which mediates the recruitment of bone marrow–derived cells (BMDCs) through both HIF-1α–dependent and –independent pathways (26). Therefore, alleviating radiation-induced vascular dysfunction within the TME is of great significance for enhancing the therapeutic efficacy of radiotherapy.

### 2.2 Effects of radiotherapy on the extracellular matrix

Radiotherapy-induced chronic inflammatory responses often manifest as activation and autocrine signaling of stromal cells, such as myofibroblasts, which are key drivers of fibrosis and tissue remodeling (27). Similar to microvascular injury triggered by radiotherapy, the preservation of normal fibroblast function is crucial for maintaining extracellular matrix homeostasis and improving patient prognosis (28). CAFs are the predominant population of abnormal stromal fibroblasts within the tumor microenvironment (29). The phenotypes of CAFs can vary depending on tumor type and stage, and it remains uncertain whether they exert tumor-promoting or tumor-suppressive effects (30).

During radiotherapy, CAFs are inadvertently activated and acquire tumor-promoting properties by expressing transforming growth factor β (TGF-β), a major effector molecule in both fibroblast activation and CAF differentiation (31, 32). TGF-β is a key regulator of CAF differentiation and is similar to chemokines such as CXCL12 in promoting a self-sustaining signaling loop that activates tumor cells during progression (33). Upon activation, CAFs can also interact with other TME-resident cells, such as epithelial cells and macrophages, to further promote tumor-supportive phenotypes (34). Radiation regulates the expression of integrins (α2, β1, and α5), which are essential for maintaining CAF adhesion to tumor cells, thereby impeding CAF migration and invasion. Notably, integrin β1 signaling is a major mediator of radiation resistance via the ECM (35).

Upon radiation exposure, integrin β1 forms complexes with focal adhesion kinase (FAK), a cytoplasmic tyrosine kinase that serves as a central node in integrin-mediated mechanotransduction (36, 37). The integrin β1/FAK axis activates downstream signaling cascades such as the PI3K/AKT and MAPK/ERK pathways, which confer survival advantages to tumor cells by enhancing anti-apoptotic signaling and promoting DNA double-strand break repair (38–40). Moreover, FAK activation reinforces cell–ECM adhesion and cytoskeletal stability, protecting tumor cells from anoikis and facilitating resistance to radiation-induced stress (41, 42). Importantly, integrin β1–FAK signaling also modulates ECM stiffness and organization through upregulation of fibronectin and collagen deposition, thereby reinforcing a pro-fibrotic and therapy-resistant microenvironment (43, 44). These findings highlight the therapeutic potential of integrin or FAK inhibitors in combination with RT to sensitize tumors and disrupt TME-mediated protection. Radiation-induced upregulation of matrix-associated proteins facilitates the attachment of newly synthesized ECM components, which in turn promotes radiation-induced CAF retention and fibrosis development (45). In summary, the fibrotic response to radiation is largely driven by the interaction among integrin signaling, activated tumor cells, stromal fibroblasts, and the surrounding ECM. This mechanism may underlie radiation resistance in tumors with strong fibrotic characteristics.

### 2.3 Effects of radiotherapy on the immune system

Tumor cells evade immune recognition and attack by developing various escape mechanisms, and reprogramming themselves to survive in the tumor microenvironment, which is often characterized by inflammation and immunosuppression, ultimately leading to tumor immune escape (46, 47). Radiotherapy activates reactive oxygen species (ROS) and nuclear factor kappa-B (NF-κB), which regulate the expression of proinflammatory mediators such as interleukin-1 (IL-1) and tumor necrosis factor-α (TNF-α), as well as adhesion molecules like intercellular adhesion molecule-1 (ICAM-1) and vascular cell adhesion molecule-1 (VCAM-1), thereby recruiting immune cells (48). ROS can also activate the TNF signaling pathway, triggering NF-κB activation, leading to a feedback loop that promotes TNF-α secretion (49, 50). Meanwhile, radiotherapy may simultaneously promote the recruitment of immunosuppressive cells such as tumor-associated macrophages (TAMs), myeloid-derived suppressor cells (MDSCs), and regulatory T cells (Tregs), which are relatively resistant to radiation (51, 52). Radiation-induced tumor DNA damage and fragmentation expose damage-associated molecular patterns (DAMPs), which are recognized by pattern recognition receptors (PRRs) and trigger immunogenic cell death (ICD) (53, 54). This form of cell death can elicit a potent anti-tumor immune response. Radiotherapy-induced ICD also transforms cellular factors within the TME into immune-stimulatory signals, activating dendritic cells (DCs) and promoting their maturation into antigen-presenting cells (APCs), thereby enhancing T cell activation and expansion for effective anti-tumor responses (55). Additionally, radiotherapy reshapes the immune landscape by activating tumor-reactive T cells and inducing type I interferons, particularly interferon-β (IFN-β), which synergistically augment antigen presentation and foster the infiltration and proliferation of cytotoxic CD8^+^ T lymphocytes, collectively contributing to more durable tumor control (56–58).

**Figure 1:**
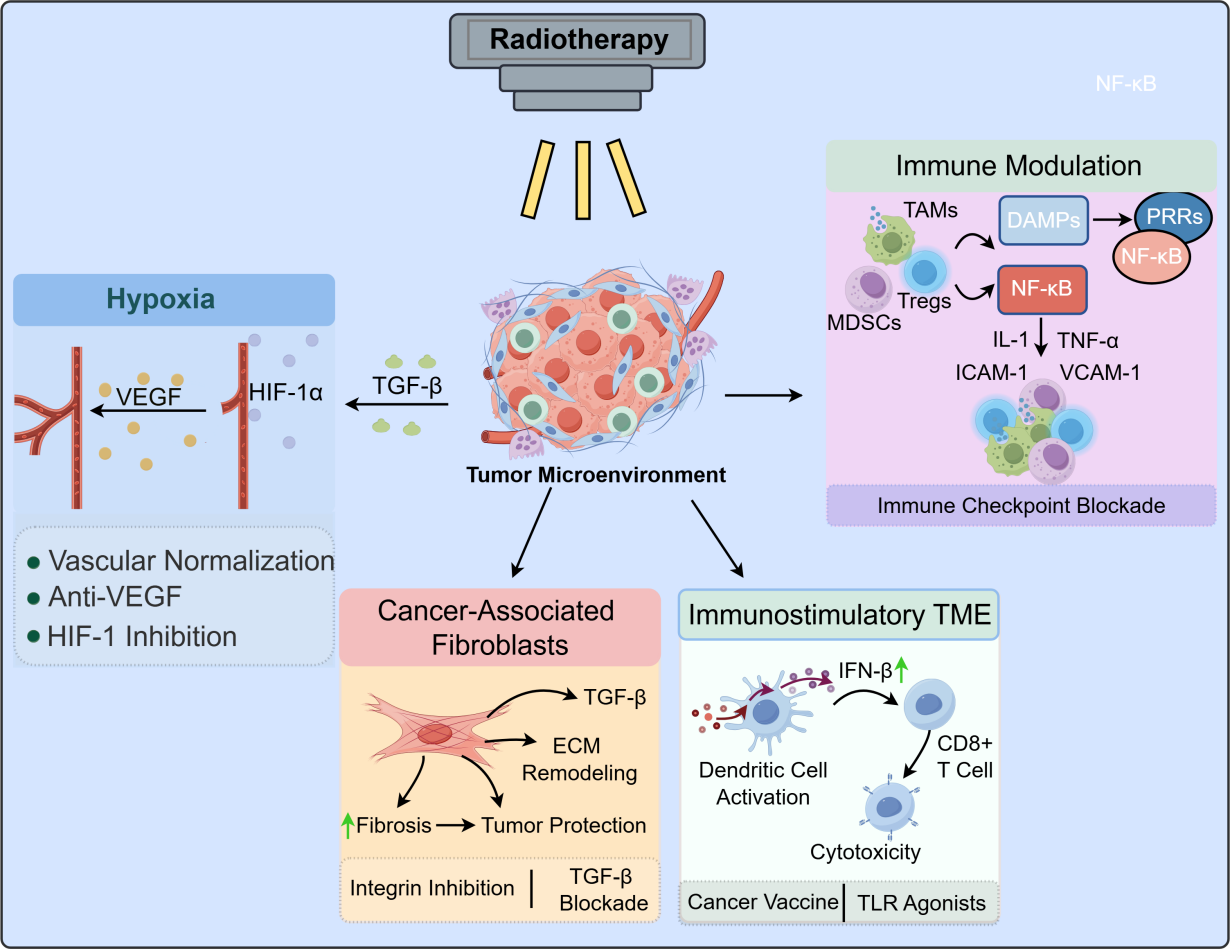
Mechanisms of the tumor microenvironment in regulating radiotherapy response.

Notably, radiotherapy does not always activate immune responses mediated by the tumor microenvironment. Although radiotherapy can induce immunogenic cell death (ICD), some tumors are still capable of evading anti-tumor immunity by activating downstream signaling pathways through autocrine or paracrine mechanisms, thereby accelerating tumor recurrence (59, 60). Besides, radiotherapy can recruit immunosuppressive cells such as MDSCs and TAMs, which secrete IL-6, IL-10, and TGF-β to inhibit T cell-mediated anti-tumor immunity (48, 51). Nevertheless, radiotherapy can upregulate the expression of co-inhibitory molecules on T cells, including cytotoxic T-lymphocyte-associated protein 4 (CTLA-4), which suppresses T cell activation by competitively binding to CD28 ligands CD80 (B7-1) and CD86 (B7-2), thereby downregulating T cell cytotoxicity and facilitating tumor immune escape (61, 62). Therefore, the immune response following radiotherapy within the TME is complex and may not be exclusively immunogenic or immunosuppressive. Radiation can alter the composition and function of immune-related factors, promote dendritic cell maturation and T cell recruitment, or conversely lead to immunosuppressive signaling pathways (6, 63). Understanding how to reshape the TME toward an immunostimulatory phenotype—transforming a “cold” tumor into a “hot” one and inducing localized and systemic anti-tumor immunity—represents a promising strategy for future radiotherapy-based tumor treatments (64–66) (**Figure 1**).

## 3 Radiotherapy resistance mediated by the tumor microenvironment

Despite numerous proposed strategies to overcome tumor radioresistance, our understanding of the mechanisms underlying TME-mediated radioresistance and potential escape pathways remains limited. Currently, therapies targeting the TME have shown promising antifibrotic and antitumor effects. However, whether these therapies can effectively alleviate TME-induced immunosuppression or prevent tumor recurrence and metastasis after radiotherapy—and whether they can ameliorate adverse post-treatment responses in patients—remains unclear. Based on the characteristics of TME remodeling post-radiotherapy, several therapeutic perspectives are proposed to potentially overcome TME-mediated radioresistance (67, 68).

### 3.1 Targeting hypoxia

Hypoxia is a critical regulator of tumor progression and has long been recognized as a major contributor to radiotherapy resistance. High tumor cell proliferation rates and abnormal vascular structures within tumors contribute to chronic, acute, and cycling hypoxia (69, 70). Radiotherapy can generate ROS that induce irreversible DNA damage in tumor cells to achieve effective tumor killing. However, radiation-induced vascular injury may exacerbate tumor hypoxia, impair oxygen diffusion, and limit the efficacy of DNA damage, ultimately leading to tumor radioresistance (71). Furthermore, tumor cells under hypoxic conditions tend to exhibit higher radioresistance and are often associated with poor prognosis in patients. Hypoxia induces upregulation of HIF-1, which regulates genes involved in tumor proliferation, metabolism, and angiogenesis. Thus, HIF-1 activity may serve as a prognostic biomarker for tumor progression and treatment resistance (72). The stability of HIF-1α in hypoxic regions is a key determinant of tumor radioresistance. Notably, HIF-1α stabilization can also be regulated through increased levels of TGF-β within the TME following radiotherapy (73, 74). Since TGF-β is also activated by radiation and promotes post-irradiation angiogenesis, directly targeting the TGF-β–HIF-1α axis may pose a significant therapeutic challenge (75). Hypoxia upregulates the expression of vascular endothelial growth factor A (VEGF-A) in endothelial cells, promoting aberrant vessel growth and exacerbating hypoxia (76).

In response to hypoxia, HIF-1α transcriptionally activates VEGF-A, which in turn promotes endothelial proliferation and neovascular sprouting (77, 78). However, the resulting vasculature is often structurally and functionally abnormal—characterized by tortuous, dilated, and leaky vessels with poor perfusion and high interstitial pressure (16, 79, 80). Following radiotherapy, this HIF-1–VEGF-driven angiogenesis is paradoxically enhanced, as radiation-induced vascular damage intensifies hypoxia, stabilizing HIF-1α and perpetuating a vicious cycle of aberrant vessel growth (81). This maladaptive remodeling not only contributes to post-RT recurrence but also hampers drug delivery and immune cell infiltration, thereby compounding therapeutic resistance (48). Recent investigations have increasingly focused on strategies to enhance radiosensitivity and mitigate tumor recurrence and metastasis by promoting the normalization of aberrant tumor vasculature (82). Both direct and indirect approaches to alleviating intratumoral hypoxia have demonstrated considerable potential in preclinical and clinical settings, particularly when administered in conjunction with radiotherapy (83–85). Nevertheless, the tumor microenvironment undergoes dynamic and often unpredictable remodeling during the course of treatment, and its intricate interplay with therapeutic agents adds further complexity.

### 3.2 Targeting cancer-associated fibroblasts

Radiotherapy activates inflammatory pathways such as NF-κB, leading to the upregulation of cytokines like IL-1β, IL-6, IL-8, granulocyte-macrophage colony-stimulating factor (GM-CSF), and cyclooxygenase-2 (COX-2), which drive immune cell recruitment and chronic inflammation (86–89). Therefore, anti-inflammatory agents targeting these pathways are being explored as radiosensitizers and have shown efficacy in preclinical settings. However, most anti-inflammatory agents also inhibit angiogenesis and DC maturation, which may compromise antitumor immunity and impair BMDC activation (90, 91). Notably, using inflammation reduction as a means to prevent tumor fibrosis may weaken the tumoricidal effects of radiotherapy (27, 92). Radiotherapy may also activate CAFs, which increase the expression of stromal cytokines like TGF-β. When TGF-β is activated post-irradiation, it contributes to tumor progression by promoting fibrosis, thereby enhancing TME remodeling and immune evasion (93). Targeting TGF-β signaling, especially in combination with radiotherapy, has been shown in preclinical models to reduce tumor growth and stromal fibrosis (93, 94). Thus, combining radiotherapy with TGF-β inhibition may mitigate CAF-driven fibrosis. CAFs, which are usually more genetically stable than tumor cells, represent an attractive therapeutic target.

Activated CAFs secrete factors such as hepatocyte growth factor (HGF) and tenascin-C (TN-C), which promote tumor progression. CAFs undergoing epithelial-mesenchymal transition (EMT) may enhance radiation resistance and promote tumor invasion (95, 96). Conversely, high TN-C–expressing CAFs or those responsive to radiotherapy may also become therapeutic targets. In fact, combining radiotherapy with TN-C–targeted therapy has demonstrated synergistic antitumor effects (97, 98). However, recent studies have shown that complete ablation of CAFs in murine pancreatic cancer models may accelerate tumor progression (99, 100). Thus, understanding the dual role of CAFs—both immunosuppressive and immune-supportive—is crucial. Therapeutic strategies that selectively inhibit pro-tumorigenic CAFs and their downstream effectors may achieve more effective outcomes than indiscriminate CAF depletion.

### 3.3 Immune modulation

Current strategies combining immune modulation with radiotherapy largely aim to overcome tumor-induced adaptive immune suppression and normalize immune activity within the TME, thereby restoring effective antitumor immunity (101, 102). Effective T cell activation requires antigen presentation and proper co-stimulation by antigen-presenting cells (APCs). Immune checkpoint molecules such as CTLA-4 and OX40, along with programmed death-1 (PD-1) and CD137, regulate this T cell activation process and are considered key nodes in radiotherapy-induced immune reprogramming (103–105). Anti-CTLA-4 monoclonal antibodies have demonstrated clinical efficacy in improving overall survival in patients with late-stage melanoma. OX40, a costimulatory receptor involved in reversing T cell exhaustion, enhances both CD4^+^ and CD8^+^ T cell activation, thereby promoting antitumor immune responses (106). OX40 activation can also stimulate low-affinity T cells, counteracting T cell tolerance. Therefore, combining OX40 agonists with anti-CTLA-4 therapy and radiotherapy holds promise for enhancing local and systemic T cell responses (107, 108). Moreover, CD137 is another potential target for co-stimulatory activation of T cells (109–111). Radiation-induced exhaustion of CD8^+^ T cells is a well-recognized mechanism of radioresistance within the TME. This exhaustion is often mediated via the upregulation of immune checkpoints such as PD-1, TIM3 (T cell immunoglobulin mucin-3), and LAG3 (lymphocyte-activation gene 3), all of which have been shown to hinder T cell responses in cancer immunotherapy (112–114).

Among these, the PD-L1/PD-1 axis is the most intensively studied. Its high expression on tumor-infiltrating lymphocytes (TILs) suggests a mechanism of adaptive immune resistance, particularly following radiotherapy (115, 116). Additionally, PD-L1 upregulation post-radiation has been implicated in acquired radioresistance (116). Studies have shown that blocking PD-L1/PD-1 signaling in combination with radiotherapy can effectively reverse resistance and enhance long-term tumor control, highlighting its potential as a therapeutic strategy in the context of immunoradiotherapy (117). Oncolytic viruses represent another promising approach for inducing ICD in tumors by preferentially infecting and lysing tumor cells. They promote DAMP release and DC recruitment within the TME, leading to robust T cell infiltration and activation (118).

Such virus-induced responses may help overcome tumor-induced immune suppression and reprogram the TME into an immunostimulatory state. In addition, studies have shown that Toll-like receptor (TLR) agonists can serve as tumor antigens or enhance innate immunity through pattern recognition pathways (119, 120). Cancer vaccines targeting TLRs have been demonstrated to effectively activate dendritic cells (DCs) and enhance T cell responses, triggering adaptive immunity in the post-radiation setting (121, 122). TLR agonists such as CpG oligodeoxynucleotides (TLR9) and poly(I:C) (TLR3) have shown potent synergistic effects with RT in preclinical models of glioma and colon cancer by promoting DC maturation and type I interferon secretion (123–125). In particular, RT-induced tumor antigen release creates a fertile immunogenic context, which TLR agonists can exploit to drive robust Th1-polarized immune responses. Preclinical evidence supports that intratumoral injection of TLR agonists, when combined with radiotherapy, can reprogram the immunosuppressive tumor microenvironment into an immune-permissive state—offering a rationale for applying this strategy to immunologically cold tumors (126, 127). Furthermore, combining GM-CSF with radiotherapy has been shown to reduce tumor metastasis and improve local tumor control, resulting in better survival outcomes for patients (128, 129) (**Table 1**).

**Table 1:** Tumor microenvironment–mediated mechanisms of radiotherapy resistance and corresponding combinatorial therapeutic strategies.

TME component	RT-induced alteration	Mechanistic consequence	Potential interventions
Vasculature	Endothelial dysfunction, increased permeability, vessel collapse, hypoxia	HIF-1α activation; recruitment of BMDCs; reduced oxygen diffusion; tumor radioresistance	Vascular normalization (anti-VEGF), HIF-1α inhibitors
Carcinoma-Associated Fibroblasts	CAF activation, TGF-β and ECM protein upregulation (e.g., TN-C), fibrosis	Fibrotic ECM remodeling, CAF-mediated immune evasion, integrin β1–mediated radioresistance	TGF-β blockade, integrin inhibitors, TN-C targeting
Extracellular Matrix (ECM)	ECM deposition, integrin upregulation, stromal stiffening	Enhanced tumor cell adhesion, reduced drug/radiation penetration	Anti-fibrotic agents, ECM remodeling strategies
Pro-tumor Immune Cells (Tregs, MDSCs, TAMs)	Selective recruitment, IL-6/IL-10/TGF-β–mediated suppression	T cell suppression, impaired cytotoxicity, immune escape	Cell depletion, reprogramming agents, immune checkpoint blockade
Anti-tumor Immune Cells (CD8^+^ T cells, DCs)	ICD, IFN-β expression, DC maturation and antigen presentation	Enhanced adaptive immunity and tumor control	ICD inducers, TLR agonists, DC vaccines, GM-CSF
Checkpoint Molecules	Upregulation of PD-L1, CTLA-4, TIM3, LAG3 on T cells	T cell exhaustion, immune suppression, immune escape	Anti-PD-1, anti-CTLA-4, OX40/CD137 agonists

## 4 Conclusion and outlook

Radiotherapy remodels the tumor microenvironment by regulating growth factors (TGF-β, VEGF, and PDGF), cytokines (CXCL12, IL-1, IL-2, IL-6, and IL-10), and transcriptional regulators (NF-κB and HIF-1), thereby influencing inflammatory responses, oxygen cycling, immune regulation, angiogenesis, and fibroblast (CAF)-mediated ECM remodeling and fibrosis. These effects can have dual outcomes: on one hand, they may enhance anti-tumor responses; on the other hand, they promote immunosuppression, leading to radioresistance, tumor recurrence, and metastasis. Against this backdrop, emerging therapeutic strategies aim to rebalance TME dynamics during and after radiotherapy, transforming tumors into more treatable forms while minimizing adverse reactions. Current strategies under development include enhancing radiosensitization through oxygen modulation, HIF-1 blockade, and vascular normalization. Integrin inhibition has also shown promise in disrupting CAF anchorage and preventing ECM-driven fibrosis and immune exclusion, making it another key direction in combination therapies.

Research on TME–radiotherapy interactions has yielded a series of promising new therapeutic strategies. Given the diverse effects of radiotherapy on the TME, the development of rational combination regimens involving radiosensitizing agents in this context will be a significant challenge. Nevertheless, we firmly believe that integrating radiotherapy with vascular normalization, immune modulation, and anti-fibrotic strategies will effectively enhance therapeutic efficacy while minimizing unnecessary side effects such as resistance and recurrence. Despite many challenges ahead, we remain optimistic that combined therapeutic strategies targeting the TME will hold broad prospects in future cancer radiotherapy.
